# ASAVACT: Arabic sentiment analysis for vaccine-related COVID-19 tweets using deep learning

**DOI:** 10.7717/peerj-cs.1507

**Published:** 2023-10-26

**Authors:** Sarah Alhumoud, Asma Al Wazrah, Laila Alhussain, Lama Alrushud, Atheer Aldosari, Reema Nasser Altammami, Njood Almukirsh, Hind Alharbi, Wejdan Alshahrani

**Affiliations:** Department of Computer Science, College of Computer and Information Sciences, Al-Imam Mohamed Ibn Saud Islamic University, Riyadh, Saudi Arabia

**Keywords:** Deep learning, Machine learning, Text mining, Natural language processing, Sentiment analysis, COVID-19 vaccine, Twitter

## Abstract

COVID-19 has become a global pandemic that has affected not only the health sector but also economic, social, and psychological well-being. Individuals are using social media platforms to communicate their feelings and sentiments about the pandemic. One of the most debated topics in that regard is the vaccine. People are divided mainly into two groups, pro-vaccine and anti-vaccine. This article aims to explore Arabic Sentiment Analysis for Vaccine-Related COVID-19 Tweets (ASAVACT) to quantify sentiment polarity shared publicly, and it is considered the first and the largest human-annotated dataset in Arabic. The analysis is done using state-of-the-art deep learning models that proved superiority in the field of language processing and analysis. The models are the stacked gated recurrent unit (SGRU), the stacked bidirectional gated recurrent unit (SBi-GRU), and the ensemble architecture of SGRU, SBi-GRU, and AraBERT. Additionally, this article presents the largest Arabic Twitter corpus on COVID-19 vaccination, with 32,476 annotated Tweets. The results show that the ensemble model outperformed other singular models with at least 7% accuracy enhancement.

## Introduction

In January 2020, the Chinese authorities announced the emergence of a new virus, COVID-19. Since then, the virus has spread rapidly worldwide, with more than 212 million positive cases and more than four million reported deaths. The pandemic has not only affected the health sector but has also affected the global economy, as well as the social and personal realms with unprecedented impact. The government authorities in the Arabian countries boarding the Persian Gulf (those share the same culture and dialect: Saudi Arabia, Kuwait, Oman, Qatar, Bahrain, and United Arab Emirates) have passed many laws and strict measures to control and reduce the effect of the virus, such as closures of businesses and social distancing; people have been affected noticeably by these measures. Hence, it has become important to understand people’s emotional responses and feelings surrounding the pandemic. This can be done in several ways; one way is to survey people directly. This is associated with the cost of contacting potential participants and gathering data. Another way is to gauge sentiment from publicly available data on social media, and then use artificial intelligence to analyze it. This could cover a larger sample with minimal analysis efforts as opposed to traditional surveys (Alhumoud et al., 2015; Alhumoud, 2018).

Twitter is used heavily in the Gulf region for communicating news from authorities and government parties. In 2021, Saudi Arabia was top Arab country with the most active Twitter users, more than 70% of the Saudi population used Twitter with more than 9.9 million active users (gmi_blogger; FinancesOnline, 2021). Several occasions proved that the public talk on Twitter indeed led to change and improvements in services in the Saudi and Gulf context.

Since the pandemic began, the governments in the Gulf region, represented by the MOH, of those countries has followed best practices, including providing the vaccine for free to all citizens and residents. Although the MOH recommended that people be vaccinated for the safety and health of the entire population, there are some people who are opposed to the vaccine.

This article aims to classifying the tweets into pro-vaccine and anti-vaccine using a state-of-the-art deep learning (DL) models, namely, the stacked gated recurrent unit (SGRU), the stacked bidirectional gated recurrent unit (SBi-GRU), and the ensemble architecture of SGRU, SBi-GRU, and AraBERT. In addition, the article aims to exploring the opinion of the Gulf region population on specific types of vaccines (Pfizer, AstraZeneca) using data mining methods.

The contribution of this study is as follows: First, present a literature review on sentiment analysis in the domain of COVID-19 vaccination. Second, compile a corpus of four million tweets related to the COVID-19 vaccine topic in Arabic; it is regarded as the largest in its genre. Third, annotate a corpus with seven language experts. Fourth, analyze the data using different DL models and an ensemble model made up of three DL models. Finally, mining the opinion of the Gulf region population regarding vaccination.

The rest of the article will include review methodology, literature review, study’s methodology, results and discussion, and finally the conclusions.

## Review Methodology

The focus of this review is on studies that utilized machine learning (ML) and DL techniques for sentiment analysis. The search sentence used is “x y z”, where x is “COVID” and y is “vaccine”, while z is “sentiment analysis” or “text analysis” or “opinion mining”. The libraries under consideration are IEEE, Springer, ACL, Microsoft Academic, MDPI, ScienceDirect, and IOP. Table 1 displays the number of results found in each database up until early April 2021. To select and narrow down the number of papers, we chose ones about or associated with Arabic sentiment analysis, that are COVID-19-related. As there are no studies on vaccine sentiment analysis in Arabic up to the study’s date, we included non-Arabic papers on vaccine-related sentiment analysis as well. All irrelevant and duplicated papers are eliminated, we ended up with a total of 11 papers.

**Table 1:** Number of papers found.

**Keywords**	**IEEE**	**Springer**	**ACL**	**Other**
COVID +Sentiment +Analysis	61	473	315	906
COVID +Text +Analysis	51	5,128	666	2,462
COVID +Opinion +Mining	14	388	253	397
COVID +Text +Mining	31	1,741	497	473
Total before elimination	157	7,730	1,731	4,238
**Total after elimination**	3	1	3	4

## Literature Review

Researchers are working to investigate efficient and accurate AI and ML models for several applications to identify, track and forecast outbreaks (Bullock et al., 2020). Studies are mainly aiming to do four of the tasks as discussed in Alhumoud (2020).

In this study, the focus is on the first task, social media analysis and sentiment analysis in specific. Mining data for meaning and trends from social media is a low-cost process that yields insight. COVID-19 related data analysis in social media is concerned with three main platforms, Twitter, Chinese’s Weibo, and Facebook. Twitter analysis was either topic modeling (Muthusami, Bharathi & Saritha, 2020; Santis, Martino & Rizzi, 2020; Singh et al., 2020; Zhao et al., 2020, p. 19; Abd-Alrazaq et al., 2020), or sentiment analysis (Mathur, Kubde & Vaidya, 2020; Manguri, Ramadhan & Mohammed Amin, 2020), or location mapping (Jahanbin & Rahmanian, 2020; Singh et al., 2020).

Studying the sentiment on COVID-19 vaccination in different parts of the world using ML is an interesting task. The literature on Arabic sentiment analysis is focused on COVID-19-related sentiment (Arabic sentiment analysis on COVID-19), while ‘Vaccine sentiment analysis’, is focused on vaccine-related sentiment analysis.

### Arabic sentiment analysis on COVID-19

This section examines six studies on Arabic sentiment analysis of COVID-19 related data using different models and approaches, including ML and DL.

Mostafa (2021) proposed a sentiment analysis model that utilizes text processing, feature selection including document frequency, skip-gram, and ML classification. Three classifiers were employed: naive bayes (NB), support vector machine (SVM), and the decision tree classifier (DTC). The dataset comprised 1,000 sentiments of students on the e-learning process during COVID-19. The dataset was divided into 700 that accepted e-learning and 300 who refused e-learning. The findings showed that the best classifier accuracy outcome was NB, with an accuracy of 87% for document frequency and 91% for the skip-gram.

Alanezi & Hewahi (2020) aimed to ascertain the impact of social distancing on people’s perspectives using Twitter sentiment analysis and clustering with k-means. They compared k-means clustering and mini-batch k-means clustering using COVID-19 related tweets. There are four datasets discussed in this article; the first two datasets from World Health Organization (WHO) and the Bahrain Ministry of health dataset are used to explore the most frequent words used. The other two datasets, the English tweets dataset and the Arabic tweets dataset have been used in applying several visualizations of k-means and Mini-Batch k-means cluster in principal component analysis (PCA) and t-distributed stochastic neighbor embedding (t-SNE). The authors showed that the United States (US) has the highest neutral tweets along with Australia, Nigeria, Canada, and the United Kingdom (UK). However, both Italy and India have the majority of positive tweets. This indicates that the people of Italy and India are more optimistic than other nations about the pandemic. Another finding is that the minibatch k-means model required less time to be built compared to the k-means with a slight difference in performance. The challenges faced by the researchers are as following: limited resources by not having access to cloud services such as Amazon Web Services (AWS). The next challenge is having noisy data where spam tweets are affecting the quality of the data and takes more time in cleaning. This could be solved by the algorithm proposed by Al Twairesh et al. (2016). However, no metrics were provided to evaluate their performance as they used visualization.

Alsudias & Rayson (2020) analyzed topics discussed on Twitter from December 2019 to April 2020. They identified and detected rumors related to COVID-19, and predicted the types of sources of tweets about COVID-19. They used n-gram forms of the Twitter corpus and clustered them using the k-means algorithm with Python Scikit. Moreover, they applied three different ML algorithms: logistic regression (LR), SVM, and NB. The dataset size is one million Arabic tweets. After applying the ML algorithms, the results show an accuracy of 84% with the LR algorithm, 81.44% with SVM, and 81.04% with the NB.

Bahja, Hammad & Kuhail (2020) gathered data on 782,391 tweets for four months in 2020. They used a combination of ML techniques to determine what Arabic speakers were tweeting about COVID-19; these techniques included LinearSVC, multinomial naive bayes(MNB), and the DTC. They built a two-stage classifier system to pinpoint relevant tweets and divided them into three categories: safety, worry, and irony. In the first stage of the classifier, the results indicate that MNB outperformed other classifiers based on the F-measure (0.85) and area under the curve (AOC, 0.94). For the next stage of the classifier, MNB again outperformed other classifiers with the F-measure (0.79) and the AOC (0.95).

Addawood et al. (2020) studied Saudi citizens’ reactions to the COVID-19 pandemic by examining Arabic tweets. The dataset contains approximately three million tweets from January to April 2020. The authors employed ML and used hashtag categorization in the SVM dataset and the NB model. The outcomes reveal that the SVM classifier was 98%, while the accuracy of the NB was 87%.

Alhumoud (2020) employed sentiment analysis involving multiple steps starting from data collection, followed by data preprocessing, and then classification. Alhumoud explored Arabic tweets to quantify the sentiment shared during the COVID-19 pandemic using a dataset consisting of 10 million Arabic tweets. Alhumoud visualized COVID-19’s influence based on ML techniques *via* the SVM model and using an ensemble model using DL techniques (Wazrah & Alhumoud, 2021). The results suggest that the ensemble model outperformed ML techniques using the SVM model with an accuracy of 90%, exceeding that of the ML with 23% in accuracy.

Among the six reviewed studies related to COVID-19 in this section, only one applied a DL model (Alhumoud, 2020). That model was proposed by the same authors of this article (Wazrah & Alhumoud, 2021).

### Vaccine sentiment analysis

In this section, we review five papers on sentiment analysis on the vaccine topics and not necessarily in Arabic, as vaccine-related sentiment analysis studies in Arabic is not available to the point of writing this article. We highlighted different aspects in those studies such as the models used to be either ML or DL, platform, dataset size, metrics, and the geographical area the study is applied in.

Li et al. (2022) carried out a number of NLP techniques and models such as TF-IDF with DT/ RF/ NB/ SVM/ LR, and FastText/ GloVe with LSTM to build a Vaccine Acceptance Index (VAI). The VAI measures the pro-vaccine and anti-vaccine opinions on COVID-19 vaccination of U.S. Twitter users. To conduct their approach, a dataset consisting of 17,000 tweets were observed throughout time to allow a deeper insight into the change in users’ opinions, this dataset was annotated and used to train and test the models. The results of their approach after several experimental methods have reached the best performance with TF-IDF + RF with an accuracy of 74.4% in the testing set. After testing the model, the model then is applied to the 29 million collected dataset. The authors reported some findings in the period from 2020 to January 2021 that the observed VAI between anti-vaccine and pro-vaccine opinions in this study has changed from anti-vaccine to pro-vaccine.

Paul & Gokhale (2020) performed sentiment analysis by examining the social parameters of tweets in the US and used them in a classification framework that harnesses different ML and DL techniques. For the framework, they started with feature extraction and included linguistic, auxiliary, social, and sentiment features. Next, they employed different ML and DL algorithms, including random forest (RF), SVM, multilayer perceptron (MLP), gradient boosting (GB), and long short-term memory (LSTM); they compared performance using the following metrics: accuracy, precision, recall, and the F-score. They also showed the results for both CBOW and skip-gram representations that they had tried for word embeddings. The classification framework can distinguish between anti-vaccine and pro-vaccine tweets with an accuracy of 83%. The percentage of classification was 66% for pro-vaccine and 34% for anti-vaccine.

To et al. (2021) evaluated the performance of different natural language processing models such as BERT and Bi-LSTM with classic ML methods, including SVM and NB, to identify anti-vaccine tweets published during the COVID-19 pandemic. The BERT model achieved high performance with 91.6% accuracy and an AUC of 84.7%, with a dataset size of 1,474,276 tweets. The percentage of anti-vaccine tweets was 9.1%; the other 90.9% were neutral, news, or ambiguous. The country this study is focused on is not refined.

Cotfas et al. (2021) aimed to analyze the dynamics of opinions on COVID-19 vaccination by considering the one-month period following the first vaccine announcement. Using Twitter, they collected and scrutinized 2,349,659 tweets and connected them to the events reported by the media. Based on analysis *via* ML, they classified tweets into three main categories: in favor, against, and neutral regarding COVID-19 vaccination. They employed BERT with an accuracy of 78.94%; the anti-vaccine class comprised 14.38% of the dataset, followed by neutral 68.90% and in favor 16.72%.

Hussain et al. (2020) developed a new hierarchical hybrid ensemble-based AI model for thematic sentiment analysis. This model utilized an average weighting ensemble of two lexicon-based methods: Valence Aware Dictionary for Sentiment Reasoning (VADER) (Hutto & Gilbert, 2014) and TextBlob library for text processing. These were combined with a pre-trained BERT model using a rule-based ensemble method. They extracted over 300,000 social media posts related to COVID-19 vaccinations, including 23,571 Facebook posts from UK and 144,864 from US, along with 40,268 tweets from UK and 98,385 from US. There was a technical limitation of their approach related to the accuracy of the AI techniques. Based on the validation results of 10% of manually annotated tweets, the final model utilized the classification output of the BERT model for neutral and negative sentiments. In contrast, the weighted-average output of lexicon-based methods was selected for classifying positive sentiments. No metrics evaluation of their performance was provided as they combined dictionary with pre-trained model for plotting temporal and geo-spatial analysis.

Table 2 outlines the vaccine-analysis-related literature. As shown below, the most commonly studied platform for vaccine-related sentiment analysis was Twitter, followed by Facebook. The most common techniques employed were ML compared to DL, and the largest dataset among the papers contained 158 million tweets (Hussain et al., 2020). Some of the papers used accuracy metrics to gauge the models’ performance. Additionally, the table shows the country of each paper.

**Table 2:** Literature review of non-Arabic sentiment analysis.

**Ref**	**Platform**	**Technique** **(ML), [DL]**	**Dataset size**	**Metrics (Accuracy)**	**Country**
Li et al. (2022)	Twitter	DT, RF, NB, SVM, LR), [LSTM]	29,213,502 Tweets	74.4%	US
Paul & Gokhale (2020)	Twitter	(RF, SVM, MLP, GB) [LSTM]	2,616 Tweets	83%	US
Cotfas et al. (2021)	Twitter	(RF, SVM) [CNN, RNN, LSTM, Bi-LSTM]	2,349,659 Tweets	78.94%	France
To et al. (2021)	Twitter	(NB, SVM) [Bi-LSTM, BERT]	1,474,276 Tweets	91.6%	NA
Hussain et al. (2020)	Facebook and Twitter	[GDPR, VADER, BERT]	158M Tweets	NA	US, UK

The methods used in the studies mentioned in this section that analyzed vaccine related sentiment are challenging to compare with the methods used in our study, as they analyzed English texts. This necessitates using different modeling techniques and different language models than the ones used in this study for analyzing Arabic texts. However, the majority of studies apply various ML techniques for classification. Thus, we will compare our model with various ML models specifically on our multi-class dataset.

## Methodology

The architecture of the proposed system passes through multiple steps starting from data collection, followed by data preprocessing, and then classification and analysis. Specifically, in this study, those steps include data collection using Twitter API, spam filtering, preprocessing, annotation, classification using SGRU, SBi-GRU, AraBERT, and the ensemble model; and finally data analysis.

**Figure 1:**
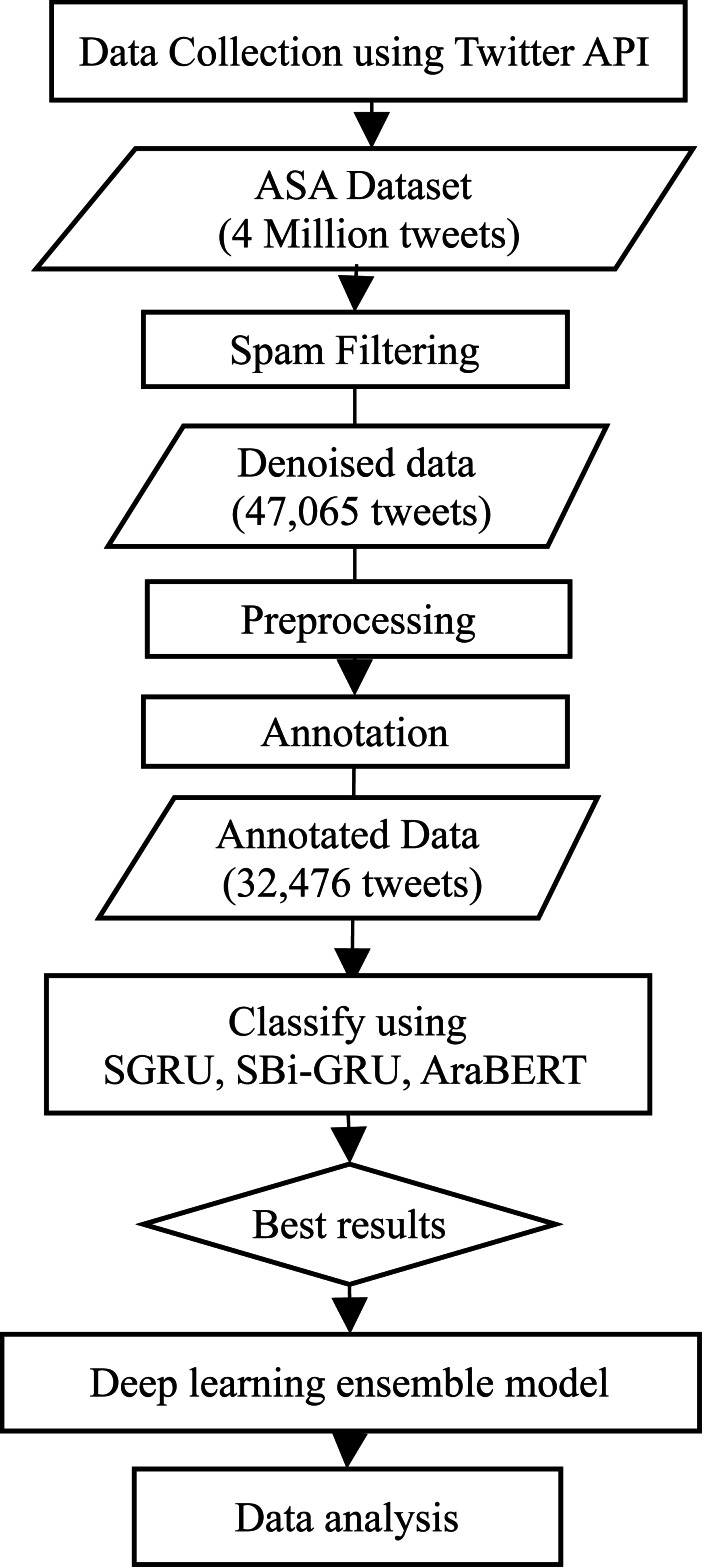
The study architecture with DL model and data analysis.

The system architecture is depicted in Fig. 1. The following sections represent the main processes in the system architecture: data collection, preprocessing, annotation, classification, and data analysis.

### Data collection

Using the Twitter streaming API and the twitterR package for R, a total of four million tweets were collected in June 2021 using 14 related keywords: { 
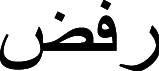
 (reject), 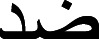 (against), 
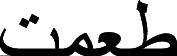
 (vaccinate), 
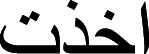
 (taken), 
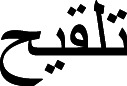
 (immunization), 
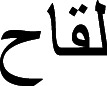
 (vaccine), 
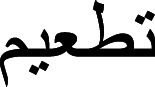
 (vaccination), 
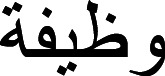
 (function), 
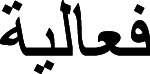
 (effectiveness), 
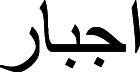
 (force), 
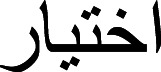
 (choice), 
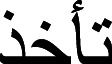
 (take), 
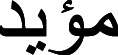
 (supporter), 
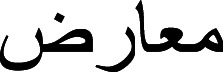
 (opposition)}. the keywords selected by observing major trends related to the topic of COVID-19 vaccine.

### Data preprocessing

Data preprocessing is necessary to minimize the noise in data and to maximize classification accuracy and success. First, tweets were cleaned from unrelated content, such as user mentions, and non-Arabic characters, except for the hashtag symbol. Additionally, punctuation, repeated letters, numbers, and extra spaces are removed. Removing stop words was postponed to after the annotation step to preserve the meaning and clarity of the sentences.

Next, we carried out spam filtering according to Al Twairesh et al. (2016), which includes deleting duplicate tweets and tweets that have one of the predefined spam keywords. The spam keyword list is from Saad (2019) which contained 258 keywords and was modified by removing 11 vaccine-related keywords and adding nine new spam keywords. In Al Twairesh et al. (2016), one of the spam conditions is having more than four hashtags in one tweet. This condition was tested in the case of the dataset under study. In specific, 4,000 random tweets were checked manually and 100% of them were vaccine-related. Thus, the condition suggested by Al Twairesh et al. (2016) was no longer valid in the case of this study. The number of remaining tweets after spam filtering decreased by 98%, leaving 47,065 tweets. Then, the tweets were cleaned of emoji and stop words. The last step was normalization, which is done by unifying multiple-form letters into one of their forms (Alhumoud, Albuhairi & Alohaideb, 2015); for example, unifying ( 
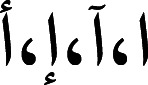
) into ( 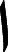).

### Dataset annotation

The ASAVACT dataset was divided into seven batches; each batch was labeled by two language experts independently. The labels were neutral, pro-vaccine, anti-vaccine, and unrelated to the topic. To decide on conflicting labels, the technique suggested by Al Twairesh et al. (2016), and Paul & Gokhale (2020) was followed. That is, tweets are eliminated if they had a labeling disagreement between at least two annotators. Based on the above, the total number of tweets that resulted after elimination was 32,476, losing 31% Of the tweets. The number of pro-vaccine tweets accounted for 38.06%, while the anti-vaccine tweets accounted for 16.64%. Neutral tweets comprised 34.83%, and unrelated tweets amounted to 10.47% as shown in Table 3.

**Table 3:** The matching results of the language experts’ annotation process after agreement.

	**Neutral (0)**	**Pro-vaccine (1)**	**Anti-vaccine (2)**	**Unrelated (3)**
**Exp 1**	16,802	18,040	7,313	4,930
**Exp 2**	17,412	17,117	7,890	4,646
**Final**	11,311	12,360	5,404	3,400

In order to measure the overall agreement and performance of the language experts, Cohen’s Kappa score is considered. Cohen’s Kappa is measured on a scale between 0 and 1, where 0 indicates no agreement, 0–0.20 indicates weak agreement, 0.21–0.40 indicates fair agreement, 0.41–0.60 indicates moderate agreement, 0.61–0.81 indicates substantial agreement, and 0.81 - 1 indicates almost perfect agreement. Cohen’s Kappa score can be computed for both binary and multiclass problems, which can be calculated from the observed agreement ratio *Po* and expected agreement ratio *Pe* as seen in (1).

(1) \begin{eqnarray*}k= \frac{{P}_{o}-{P}_{e}}{1-{P}_{e}} .\end{eqnarray*}

The observed agreement ratio *Po* is the number of labeled tweets that the language experts agreed on divided by the total number of labeled tweets. And the expected agreement ratio *Pe* is calculated by summing the probability of each label. The label probability is the total number of each label being labeled by the first language expert divided by the total number of labeled tweets, multiplied by the total number of the same label being labeled by the second language expert divided by the total number of labeled tweets (Conger, 1980). As a result, the average Cohen’s Kappa was 0.51 (51%).

### Classification

To classify the data, we used three different DL techniques: SGRU, SBi-GRU, and the ensemble model (Wazrah & Alhumoud, 2021), which are depicted in the following subsections. ‘SGRU modeling’ and ‘SBI-GRU modeling’ cover the proposed SGRU and SBi-GRU architectures. In addition, ‘AraBERT’ covers the AraBERT pre-trained language model based on Google’s BERT architecture (Devlin et al., 2019). Finally, ‘Ensemble model (AraBERT + SGRU + SBi-GRU):’ deals with the proposed ensemble model, which is based on SGRU, SBi-GRU, and AraBERT.

**SGRU modeling:** GRU is a variation of LSTM introduced by Cho et al. (2014) , which uses two gates instead of three, as in LSTM, and fewer parameters; thus, it is a simpler model. SGRU model (Wazrah & Alhumoud, 2021) is composed of several GRU units and we use the stack concept to find out the best number of layers for the model.

**SBI-GRU modeling:** Schuster and Paliwal proposed the first bidirectional recurrent model (Schuster & Paliwal, 1997). Instead of only depending on the forward elements alone as in the latter. Bi-GRU consists of two GRUs: the first GRU goes from left to right, while the other GRU does the reverse. This approach would potentially increase the amount of input information available to the network and hence generate more accurate outputs in each time step. The SBi-GRU model (Wazrah & Alhumoud, 2021) is composed of several forward layers and several backward layers stacked above each other. We use the stack concept on Bi-GRU to find out the best number of layers for the model.

**AraBERT:** AraBERT (Antoun, Baly & Hajj, 2020) is an Arabic pre-trained language model based on Google’s BERT architecture (Devlin et al., 2019). The model was trained on ∼70 million sentences or ∼23 GB of Arabic text with ∼3 billion words. AraBERT achieved state-of-the-art performance compared to other contextualized embedding (Antoun, Baly & Hajj, 2020). The performance of AraBERT is compared with that of Google’s multilingual BERT and other cutting-edge approaches, with an accuracy of 51% for the BERT model and an accuracy of 58.9% for the AraBERT model. Language-specific BERT models have recently been proven to be effective in language comprehension with the growth of transformer-based models because they are pre-trained on an incredibly broad corpus. These frameworks set high benchmarks for certain NLP activities and exhibit state-of-the-art performance (Antoun, Baly & Hajj, 2020). In this research, we investigate the model efficiency and compare its accuracy with SGRU. Moreover, in the next section, we apply the ensemble model based on AraBERT, SGRU, and SBi-GRU.

**Ensemble model (AraBERT + SGRU + SBi-GRU):** Ensemble modeling is a technique of weighing individual results and combining them to arrive at a final decision (Polikar, 2006). This technique has been successful at improving the accuracy of ML models by training several individual classifiers and combining them to improve the overall predictive power of the model (Polikar, 2006). The model utilize several classifiers by combining them in some manner to obtain the outcome, either by performing the weighted average or majority voting of the individual classifiers. This process improves the model’s overall accuracy when compared to the accuracy obtained by using a single classifier. Inspired by Wazrah & Alhumoud (2021), we employed three models—AraBERT, SGRU, and SBi-GRU—to obtain the voting of the final classification. Table 4 present the hyperparameters configuration of SGRU, SBi-GRU and AraBERT.

**Table 4:** Models’ configuration.

**Hyperparameters**	**SGRU and SBi-GRU**	**AraBERT**
**Batch size**	64	32
**Dropout**	0.2 in each layer	–
**Nodes**	100 in each layer	–
**Training split**	0.8	0.9
**Validation split**	0.1	–
**Testing split**	0.1	0.1
**Epoch**	5	6
**Optimizer**	Adam	–
**Loss function**	Categorical cross-entropy	–
**Vector size**	200	–

### Automatic sentiment refinement

The study Wazrah & Alhumoud (2021) presented automatic sentiment refinement (ASR) algorithm in which they found that removing double sentiment words increases accuracy by around 10%. The ASR defines double sentiment words as the words that appear almost equally in all classes, and removing them will aid the learning. Here in this article ASR is used with SGRU and SBi-GRU only as AraBERT is pretrained to learn from context.

## Results and Discussion

In this section, results of the DL models used in this research are depicted. Both SGRU and SBi-GRU contain stacked GRU layers and Bi-GRU layers respectively, and performance is measured while increasing the number of layers. The results are presented in the following four sections. Where the first section presents the actual sentiment results of the vaccination sentiment. The other three sections explain the results of the DL models.

### Arabic sentiment on the vaccine

**Figure 2:**
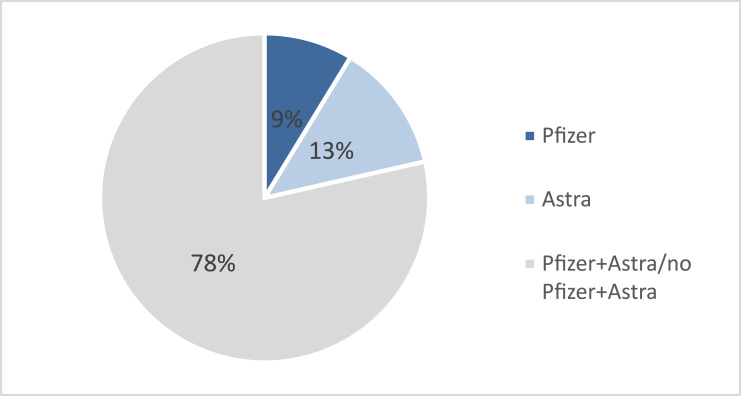
Resulted vaccine sentiment.

We analyzed the curated ASAVACT dataset of 32,476 tweets on COVID-19 vaccines. The results indicate that the majority of the tweets mentioned both vaccines, that is 78% of the tweets mentioned both Pfizer and AstraZeneca. While tweets that mentioned AstraZeneca alone are 14%, and tweets on Pfizer were 9% of total tweets, as shown in Fig. 2.

**Figure 3:**
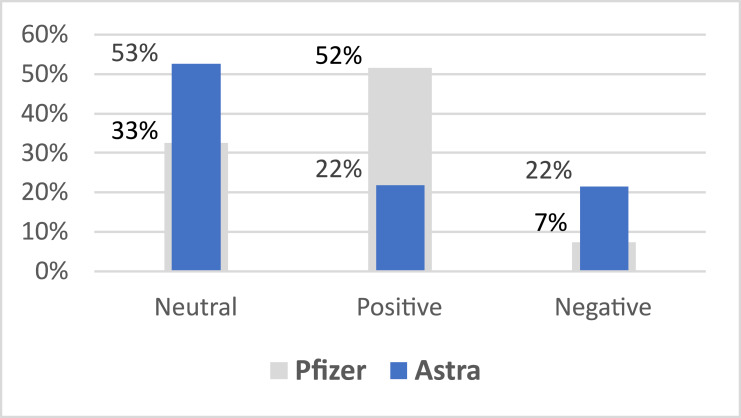
Overall averaged vaccine sentiment.

Moreover, the results indicate that most of the positive sentiments on the vaccine were related to Pfizer with 52% of the total Pfizer-related tweets are positive. Also, most of the negative sentiments were related to AstraZeneca vaccine scoring 22% compared to 7% only for Pfizer negative sentiments, as shown in Fig. 3. This could be understandable as fake news on correlations of AstraZeneca to strokes were circulating at that time.

The results also show that AstraZeneca tweets are more neutral than Pfizer, as the neutral tweets comprises 53% tweets on AstraZeneca compared to 33% on Pfizer.

**Figure 4:**
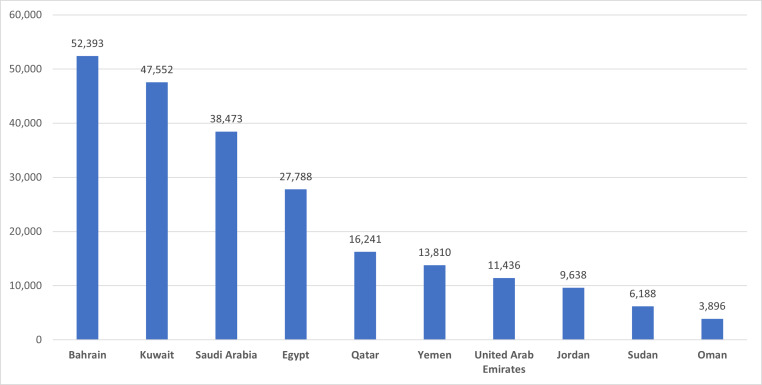
Top 10 tweeters’ countries in the ASAVACT dataset.

To study the sentiment exact location the tweets were verified against location information, only 32.22% of collected tweets have location information. The top 3 active tweeters in the Arab countries in the ASAVACT dataset were from Bahrain, Kuwait, and Saudi Arabia respectively. They all represent Gulf region, assuring the higher participation among all other Arab countries. Figure 4, shows top 10 tweeters’ countries.

### SGRU and SBI-GRU

When comparing the SGRU model with a similar SBi-GRU model, the accuracy rises when the number of layers increases. We found that the SGRU model outperformed the SBi-GRU model in terms of accuracy. In the case of SBi-GRU, the model with the highest accuracy was the architecture with five layers. The accuracy achieved was 70.47%, which is nearly equivalent to that of the SGRU (70.91%).

The increase in the number of layers resulted in additional extracted features from the model. However, we observed this pattern for a certain increase in the number of layers; then performance either became stable or decreased. The accuracy obtained was the optimum for the set of hyper parameters of the SGRU and SBi-GRU. Moreover, stacking layers perform better with the GRU than with Bi-GRU, with an accuracy of 70.91% in the three-layer GRU and an accuracy of 70.47% in the five-layer Bi-GRU.

Comparing SGRU and SBi-GRU with various ML models such as NB, SVM, RF, LR, and DT, we found that the difference between DL and ML models are observable, with the superiority of DL in terms of textual sentiment analysis. Although there are various morphological and syntactical challenges in Arabic language, recurrent neural network based models have shown slight improvement in capturing both semantic and syntactic information without explicitly knowing the language.

Table 5 presents the classification results of SGRU, SBi-GRU and ML models.

**Table 5:** SGRU, SBI-GRU and ML models results.

**Model**	**Accuracy**	**F1**
**GRU** 1-L	70.60%	67.42%
**SGRU** 2-L	69.74%	68.20%
**SGRU** 3-L	70.91%	68.70%
**SGRU** 4-L	70.66%	68.22%
**SGRU** 5-L	70.23%	67.48%
**SGRU** 6-L	70.44%	67.15%
**Bi-GRU** 1-L	68.35%	70.60%
**SBi-GRU** 2-L	69.37%	69.05%
**SBi-GRU** 3-L	69.58%	68.38%
**SBi-GRU** 4-L	68.93%	68.16%
**SBi-GRU** 5-L	70.47%	67.67%
**SBi-GRU** 6-L	68.63%	67.54%
**SVM**	62.46%	65.13%
**DT**	53.38%	53.53%
**NB**	58.06%	63.38%
**RF**	61.57%	63.18%
**LR**	62.00%	64.38%

### AraBERT

As seen in Table 6, the AraBERT model outperformed the SGRU and SBi-GRU models in terms of accuracy when compared to the single predicting model because it uses the excellent representational power of transformers (Devlin et al., 2019). In addition, AraBERT has proven to be efficient at language understanding, because it is pre-trained on a large corpus.

**Table 6:** AraBERT and ensemble model results.

	**Accuracy**	**F1**
**AraBERT**	74.18%	69.52%
**Ensemble**	81.67%	77.12%

### Ensemble

Ensemble methods are successful because they combine the advantages of different classifiers. The ensemble model created is more accurate than its individual components. Regarding the ensemble of the best models, this model (Wazrah & Alhumoud, 2021) achieved 81% accuracy, as depicted in Table 5. We used the same BERT-Base configuration for AraBERT. The assembled methods help to reduce factors such as unwanted errors.

## Conclusions

This research presents four main contributions to the field of Arabic sentiment analysis using DL. First, a review on sentiment analysis in the domain of COVID-19 vaccination using both ML and DL models. Consequently, the need for Arabic vaccine-sentiment analysis is evident. Hence, the second contribution with the collection of 4 million tweets related to the COVID-19 vaccine topic in Arabic, which is the largest in its genre. Third, the curation and annotation of a corpus that contain 32,476 tweets, recruiting seven language experts. Fourth, the implementation of state-of-the-art DL models to analyze the corpus. In addition to proving an experiment on the shortage of including negation as a feature for Arabic sentiment analysis. The findings of the experiment indicate that the ensemble model outperformed the other singular models, scoring 81.67%, with improvements of 10.76%, 11.20%, and 7.49% over SGRU, SBi-GRU, and the ensemble model, respectively.

## Supplemental Information

10.7717/peerj-cs.1507/supp-1 Supplemental Information 1 Raw Dataset Tweet IDs

## Additional Information and Declarations

### Data Availability

The following information was supplied regarding data availability: The raw dataset (tweet IDs) are available at GitHub, Zenodo, and in the Supplemental Files: -https://github.com/imamu-asa/ASA/tree/main/ASAVACT
- Alhumoud, Sarah, Al Wazrah, Asma, Alhussain, Laila, Alrushud, Lama, Aldosari, Atheer, Altammami, Reema, Almukirsh, Njood, Alharbi, Hind, & Alshahrani, Wejdan. (2023). ASAVACT repository. Zenodo. https://doi.org/10.5281/zenodo.8313042
The code is available at GitHub and Zenodo: -https://github.com/imamu-asa/ASA/tree/main/Proposed%20models
-Alhumoud, Sarah, Al Wazrah, Asma, Alhussain, Laila, Alrushud, Lama, Aldosari, Atheer, Altammami, Reema, Almukirsh, Njood, Alharbi, Hind, & Alshahrani, Wejdan. (2023). ASAVACT repository. Zenodo. https://doi.org/10.5281/zenodo.8313042
